# Efficacy of traditional Chinese exercise for sarcopenia: A systematic review and meta-analysis of randomized controlled trials

**DOI:** 10.3389/fnins.2022.1094054

**Published:** 2022-12-22

**Authors:** Kun Niu, Ying-Lian Liu, Fan Yang, Yong Wang, Xia-Zhi Zhou, Qing Qu

**Affiliations:** ^1^College of Traditional Chinese Medicine, Hainan Medical University, Haikou, China; ^2^Department of Neurology, Minhang Hospital, Fudan University, Shanghai, China; ^3^Department of Massage, Hangzhou Hospital of Traditional Chinese Medicine Affiliated to Zhejiang Chinese Medical University, Hangzhou, China

**Keywords:** traditional Chinese exercise, traditional Chinese medicine, sarcopenia, systematic review, meta-analysis

## Abstract

**Objective:**

To conduct a systematic review and meta-analysis to evaluate the effectiveness of Traditional Chinese Exercise (TCE) for sarcopenia.

**Methods:**

A literature search was conducted in eight online databases from inception until September 2022. Based on the Cochrane risk of bias tool, randomized controlled trials (RCTs) with RoB score ≥ 4 were included for further analyses. The primary outcome was muscle strength and physical function, and the secondary outcomes were adverse events. Data collection and analyses were conducted by RevMan 5.4 Software. GRADE system was used to evaluate the certainty of evidence.

**Results:**

A total of 13 eligible RCTs with 718 subjects were identified and included in this study. Among them, 10 RCTs involved Yijinjing; 2 involved Tai Chi; and 1 involved Baduanjin. Meta-analyses showed that TCE had better clinical effects than control measures in the chair stand test (*P* < 0.00001, I^2^ = 38%; Certainty of evidence: Moderate), squatting-to-standing test (*P* < 0.00001, I^2^ = 0%; Certainty of evidence: Moderate), 6-m gait speed (*P* < 0.00001, I^2^ = 13%; Certainty of evidence: Moderate), Time Up and Go Test (*P* = 0.03, I^2^ = 81%; Certainty of evidence: Low), peak torque of the extensors (*P* = 0.03, I^2^ = 0%; Certainty of evidence: Moderate), total work of the extensors (*P* = 0.03, I^2^ = 35%; Certainty of evidence: Moderate), peak torque of the flexors (*P* = 0.03, I^2^ = 47%; Certainty of evidence: Low), total work of the flexors (*P* = 0.02, I^2^ = 42%; Certainty of evidence: Low), the average power of the flexors (*P* = 0.03, I^2^ = 30%; Certainty of evidence: Moderate), and balance function (*P* < 0.00001, I^2^ = 53%; Certainty of evidence: Low). In additional, no adverse events were reported in participants who receive TCE.

**Conclusion:**

The findings of the present systematic review, at least to a certain extent, provided supporting evidence for the routine use of TCE for sarcopenia.

## Introduction

Sarcopenia, a skeletal muscle disorder, is related to the accelerated loss of physical function and muscle mass (Cruz-Jentoft and Sayer, 2019). It is a progressive and generalized disease that is common in the elderly and is associated with various adverse outcomes including fall down, functional decline, and bodily weakness (Cruz-Jentoft et al., 2019). It severely affects the normal physiological function and quality of life of the elderly, and even shortens their lifespan (Mohd Nawi et al., 2019). In recent years, the aging of the population has become a serious social problem all over the world, and sarcopenia has received increasing attention (Jensen et al., 2020). Exercise, nutrition, and pharmacotherapy are the mainstays of treatment for sarcopenia in the elderly (Cruz-Jentoft et al., 2014). There is currently no specific cure for sarcopenia. Some drugs may benefit muscles, such as hormones, but these drugs may cause serious adverse effects (Gaskin et al., 2003; Veldhuis et al., 2011). Exercise therapy is regarded as one of the major means of treating sarcopenia in the elderly, mainly including resistance exercise and aerobic exercise (Kakehi et al., 2022).

Traditional Chinese exercise (TCE) is a therapeutic, aerobic, and mind-body exercise, which originated from traditional Chinese medicine and can be traced back to approximately 3,000 years ago (Zhang et al., 2017). As a major integral part of non-pharmacological traditional Chinese medicine, TCE mainly includes Yijinjing, Tai Chi, Baduanjin, and Wuqinxi, and are characterized by gentle movements emphasizing physical and mental relaxation (Zhou et al., 2019; Zeng et al., 2020). Previous studies had reported the significant effects of TCE in improving patients’ physical status in various diseases including metabolic diseases (Zou et al., 2019), degenerative diseases (Fidan et al., 2019), cardiovascular diseases (Wu et al., 2020), respiratory disease (Reychler et al., 2019), endocrinopathies (Meng et al., 2018), and cancer (Wayne et al., 2018).

Currently, increasing numbers of clinical trials have reported that TCE has been used for treating sarcopenia. More and more randomized controlled trials (RCTs) demonstrated that TCE can significantly improve patients’ physical status (Zhu Y. et al., 2019). However, results from different studies are inconsistent, and sometimes are contrary due to different sample sizes or duration time. The conclusions from current studies have remained controversial, and the evidence provided by these studies are require assessment. Therefore, it is worth undertaking a systematic review and meta-analysis to investigate the effectiveness of TCE for patients with sarcopenia.

## Methods

The present study is reported based on the Preferred Reporting Items for Systematic Reviews and Meta-Analyses: The PRISMA Statement (Moher et al., 2009).

### Database and search strategies

We searched four international online databases (PubMed, EMBASE, Cochrane library, and Web of Science) and four Chinese online databases (VIP information database, Chinese National Knowledge Infrastructure (CNKI), Wan Fang Data Information Site, and Chinese Biomedical Literature Database) from inception to September 2022. Additionally, other relevant studies including references cited by previously published systematic reviews, conference proceedings, and dissertations were also manually searched in this study. The following search strategy was used for PubMed and was modified to suit other databases.

1. Traditional Chinese exercise
2. Qigong
3. Tai Chi
4. Yijinjing
5. Baduanjin
6. Kungfu
7. Wuqinxi
8. OR/1-7
9. Sarcopenia
10. Sarcopenias
11. OR/8-9
12. 8AND11

### Eligibility criteria

#### Types of studies

In the present study, we only included RCTs that evaluate the efficacy and safety of TCE for sarcopenia. As some studies used the birthday, ID number, or hospitalization number as the basis for random generation, these Quasi-RCTs studies were excluded. There is no limitation on language, blinding, or publication type of included studies.

#### Types of participants

All participants with a diagnosis of sarcopenia met one of the following criteria: (i) established definition of sarcopenia by the European Working Group on Sarcopenia in Older People (EWGSOP) (Cruz-Jentoft et al., 2010); (ii) established definition of sarcopenia by Roubenoff (2000); (iii) established definition by the Asia Working Group for Sarcopenia (AWGS) (Chen et al., 2014). Other diagnostic criteria with comparable definitions were also used.

#### Types of interventions

Traditional Chinese exercise monotherapy was used in the treatment groups. There is no limitation on the frequency, intensity, or course of TCE. The comparator was one of the followings: no training or health education. The included studies should include one of the following comparisons: (1) TCE *vs.* no training; (2) TCE *vs.* health education.

#### Types of outcome measures

The primary outcome was muscle strength and physical function, which was assessed by different measures including the Grip Strength Test, Chair Stand Test, Squatting-To-Standing Test, 6-m gait speed, Peak Torque of muscle, Total Work of muscle, Average Power of muscle, Timed-Up-and-Go Test, Berg Balance Scale. All of the outcome measurements were conducted at the endpoint of treatment by the researchers in each trial.

### Study selection and data collection

Two investigators of our group selected the potential references by screening the title and abstract of each article. For those potentially eligible studies, full articles were downloaded from databases. The two investigators read the whole article independently and made the final decision on including the articles or not. For each eligible study, the following information was collected: the first author’s name and year of publication, final diagnosis, diagnosis criteria, study design, sample size, gender composition, the mean age of participants, interventions, duration of treatment, follow-up, main outcome measures, and its corresponding *p*-value. If the necessary data were expressed graphically or not recorded in the manuscripts, we tried to contact the original author for further information by phone or email or calculated by ourselves if available. Any disagreement between the two investigators was resolved through a discussion with the third author.

### Risk of bias

We assessed the methodological quality of the RCTs included in the present study with the seven criteria recommended by the Cochrane Collaboration (Cumpston et al., 2019). The seven components were as follows: A. adequate sequence generation; B. concealment of allocation; C. blinding (participants and personnel); D. blinding (outcome assessor); E. incomplete outcome data addressed (ITT analysis); F. selective reporting; G. other biases. Each of these indicators was categorized as high risk of bias, low risk of bias, and unclear. For each item, a score of 1 or 0 was given depending on whether the study provided adequate information in the relevant domain. Only RCTs with a cumulative score of at least 4 out of 7 for the Cochrane RoB tool domains were included in this systematic review. Adequate sequence generation must achieve status as low risk of bias as it is the certain key criteria. Disagreements were settled by a discussion with the corresponding author.

### Grading the certainty of the evidence

The updated GRADE system (Guyatt et al., 2013) was applied to assess the certainty of evidence using four grades: high, moderate, low, and very low. The low and very low certainty of evidence means that the true effect is likely to be substantially different from the estimate of effect, and we have little or very little confidence in the effect estimate. Any discrepancy about grading the certainty of the evidence was resolved through discussion with the corresponding author.

### Data synthesis and analysis

The software Cochrane Collaboration Review Manager (RevMan 5.4) was used to summarize the data of eligible studies and performed meta-analysis. Weighted mean difference (WMD) was adopted to analyze the continuous data, and risk ratio (RR) was adopted to analyze the dichotomous data. The standard chi-square test and I^2^ statistic were used to evaluate heterogeneity among trials. A fixed effect model or a random effect model was used to analyze pooled effects depending on heterogeneity. When there is no obvious heterogeneity, a fixed effect model was used (*P* > 0.1, I^2^ < 50%), otherwise, the random effect model was applied. Subsequent sensitivity analyses were used to explore the possible sources of heterogeneity. A probability value of *P* < 0.05 was considered significant.

## Results

### Description of studies

**FIGURE 1:**
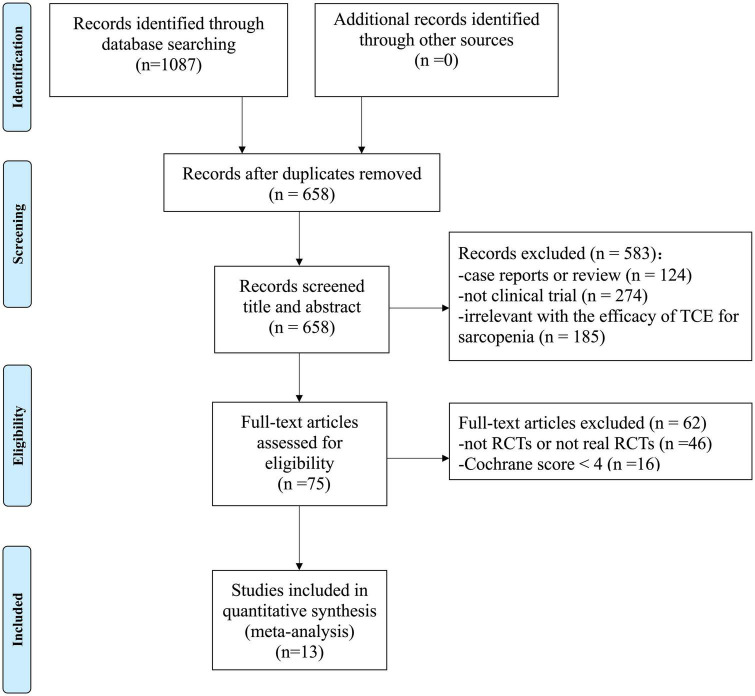
PRISMA 2009 flow diagram.

A total of 1,087 studies were retrieved, of which 659 studies remained after excluding duplicates. After screening the title and abstract of the remaining studies, 583 studies were excluded; among which 124 studies were case reports or reviews, 274 were not clinical trials and 185 were irrelevant with the efficacy of TCE for sarcopenia. By reading the full text, 62 studies were excluded, including 46 studies that were not RCTs or not real RCTs, 16 that were high risk of bias studies with Cochrane score < 4. Eventually, 13 studies (Gong et al., 2011; Jin et al., 2011; Liu et al., 2012; Liu et al., 2014; Liu et al., 2016; Wang et al., 2016; Zhao et al., 2016; Zhu et al., 2016; Zhu et al., 2017; Zhu G. et al., 2019; Zhu Y. et al., 2019; Fang et al., 2020; Zhou et al., 2020; Peng et al., 2022) with Cochrane RoB score ≥ 4 were included in the present study. The process of screening is presented in a PRISMA flow chart (Figure 1).

### Study characteristics

The detailed characteristics of the included 13 studies were summarized in Table 1. All eligible RCTs were conducted in China and 2 (Zhu et al., 2016; Zhu et al., 2017) of them were published in the English language. The diagnosis criteria included the established definition of sarcopenia reported by the EWGSOP, Roubenof et al., and the AWGS. The sample size of the included studies ranged from 12 to 77, enrolling a total of 718 participants, including 356 patients in treatment groups and 362 patients serving as controls. Comparisons of TCE therapies versus no training were conducted in seven studies [(Gong et al., 2011; Jin et al., 2011; Liu et al., 2016; Wang et al., 2016; Zhao et al., 2016; Fang et al., 2020; Peng et al., 2022)], while TCE therapies versus health education were conducted in six studies (Liu et al., 2012; Liu et al., 2014; Zhu et al., 2016; Zhu et al., 2017; Zhu G. et al., 2019; Zhu Y. et al., 2019; Zhou et al., 2020). As for interventions, Yijinjing was used in 10 studies (Gong et al., 2011; Jin et al., 2011; Liu et al., 2012; Liu et al., 2014; Liu et al., 2016; Wang et al., 2016; Zhao et al., 2016; Zhu et al., 2017; Fang et al., 2020; Peng et al., 2022), Baduanjin was used in 1 study (Zhou et al., 2020), and Tai chi was used in 2 studies (Zhu et al., 2016; Zhu Y. et al., 2019). The treatment duration ranged from 8 weeks to 18 months, and 8 weeks was used most widely. No study mentioned the length of follow-up. The outcomes index included the Grip strength test, chair stand test, squatting-to-standing test, 6-m gait speed, Time Up and Go Test, Isokinetic muscle strength test, balance function, and adverse effect.

**TABLE 1:** Characteristics of included studies.

No.	References	Final diagnosis	Eligibility criteria	Study designs	Sample and characteristics (male/female; mean age)		Interventions		Course of treatment	Follow up	Outcome index	Intergroup differences
					Trial	Control	Trial	Control				
1	Wang et al., 2016	Sarcopenia	Roubenoff’s view	RCT	38(15/23) 66.79 ± 4.76	37(7/30) 65.59 ± 3.59	Yijinjing	No training	12w	NR	Grip Strength Test Chair Stand Test Squatting-To-Standing Test	1. *P* > 0.05 2. *P* < 0.05 3. *P* > 0.05
2	Jin et al., 2011	Sarcopenia	Roubenoff’s view	RCT	26(14/22) 68.22 ± 4.09	35(7/28) 65.09 + 3.95	Yijinjing	No training	8w	NR	6-m gait speed Chair Stand Test Squatting-To-Standing Test	1. *P* < 0.05 2. *P* < 0.05 3. *P* < 0.05
3	Gong et al., 2011	Sarcopenia	Roubenoff’s view	RCT	30(7/23) 66.4 ± 5.47	30(9/21) 67.0 ± 5.28	Yijinjing	No training	8w	NR	Peak Torque Total Work Average Power	1. *P* < 0.05 2. *P* < 0.05 3. *P* < 0.05
4	Zhao et al., 2016	Sarcopenia	AWGS	RCT	6 67.8 ± 3.8	6 66 ± 3.11	Yijinjing	No training	8 w	NR	Grip Strength Test 6-m gait speed	1. *P* < 0.05 2. *P* < 0.05
5	Peng et al., 2022	Sarcopenia	AWGS	RCT	39 (17/23) 72.12 ± 6.47	38 (16/24) 71.85 ± 5.73	Yijinjing	No training	8 w	NR	Berg Balance Scale 6-m gait speed	1. *P* < 0.05 2. *P* < 0.05
6	Liu et al., 2016	Sarcopenia	Roubenoff’s view	RCT	31 (12/19) 67.86 ± 6.86	30 (12/18) 69.10 ± 6.69	Yijinjing	No training	8 w	NR	Peak Torque Total Work Average Power	1. *P* < 0.05 2. *P* < 0.05 3. *P* < 0.05
7	Fang et al., 2020	Sarcopenia	EWGSOP	RCT	18 (5/13) 82.8 ± 8.5	18 (7/11) 76.3 ± 9.9	Yijinjing	No training	6 m	NR	TUGT	1. *P* < 0.05
8	Liu et al., 2014	Sarcopenia	Roubenoff’s view	RCT	31 (12/19) 67.86 ± 6.86	30 (12/18) 69.10 ± 6.69	Yijinjing	Health education	8 w	NR	Balance Test Adverse effect	1. *P* < 0.05 2. *P* > 0.05
9	Liu et al., 2012	Sarcopenia	Roubenoff’s view	RCT	31 (12/19) 67.86 ± 6.86	30 (12/18) 69.10 ± 6.69	Yijinjing	Health education	8 w	NR	Adverse effect	1. *P* > 0.05
10	Zhu et al., 2017	Sarcopenia	AWGS	RCT	32 (17/15) 65.6 ± 11.4	31 (15/16) 66.3 ± 10.8	Yijinjing	Health education	12 w	NR	Grip Strength Test Chair Stand Test Squatting-To-Standing Test	1. *P* < 0.05 2. *P* < 0.05 3. *P* < 0.05
11	Zhou et al., 2020	Sarcopenia	AWGS	RCT	20 (8/12) 72.67 ± 9.56	20 (9/11) 73.25 ± 8.54	Baduanjin	Health education	8 w	NR	Grip Strength Test Chair Stand Test Berg Balance Scale TUGT	1. *P* < 0.05 2. *P* < 0.05 3. *P* < 0.05 4. *P* < 0.05
12	Zhu Y. et al., 2019	Sarcopenia	AWGS	RCT	24 88.8 ± 3.7	27 87.5 ± 3.0	Tai Chi	Health education	8 w	NR	Grip Strength Test 6-m gait speed TUGT Chair Stand Test Berg Balance Scale	1. *P* > 0.05 2. *P* < 0.05 3. *P* < 0.05 4. *P* < 0.05 5. *P* < 0.05
13	Zhu et al., 2016	Sarcopenia	AWGS	RCT	30 (10/20) 64.0 ± 3.0	30 (13/17) 64.0 ± 4.0	Tai Chi	Health education	18m	NR	TUGT Chair Stand Test Berg Balance Scale	1. *P* < 0.05 2. *P* < 0.05 3. *P* < 0.05

RCT, randomized controlled trials; TUGT, timed-up-and-go test; NR, not report; w, week; m, month.

### Risk of bias

The assessment information of RoB is presented in Table 2. Of the 13 included studies, 1 met six Cochrane criteria (Fang et al., 2020), 1 met five (Liu et al., 2012), and 11 met four (Gong et al., 2011; Jin et al., 2011; Liu et al., 2014; Liu et al., 2016; Wang et al., 2016; Zhao et al., 2016; Zhu et al., 2016; Zhu et al., 2017; Zhu Y. et al., 2019; Zhou et al., 2020; Peng et al., 2022). All 14 included studies had random allocation using a random number table. Only 1 study (Fang et al., 2020) mentioned allocation concealment with sealed envelopes. 2 studies (Liu et al., 2012; Fang et al., 2020) mentioned the blinding of outcome assessment. All studies either had complete data or had dropouts with adequate explanations and appropriate methods to treat missing data. All studies had a low risk of other biases, which included funding bias, conflict of interest, and incomparable baseline characteristics between the groups. Funding bias means that the research was funded by relevant stakeholders, such as drug companies. In general, most of the 14 trials were deemed to have a relatively moderate risk.

**TABLE 2:** Risk of bias.

References	7-item criteria							
	A	B	C	D	E	F	G	T
Fang et al., 2020	**+**	+	?	+	**+**	**+**	**+**	6
Gong et al., 2011	**+**	?	?	?	**+**	**+**	**+**	4
Jin et al., 2011	**+**	?	?	?	**+**	**+**	**+**	4
Liu et al., 2012	**+**	?	?	+	**+**	**+**	**+**	5
Liu et al., 2014	**+**	?	**?**	**?**	**+**	**+**	**+**	4
Liu et al., 2016	**+**	?	?	?	**+**	**+**	**+**	4
Peng et al., 2022	**+**	?	?	?	**+**	**+**	**+**	4
Wang et al., 2016	**+**	?	?	?	**+**	**+**	**+**	4
Zhao et al., 2016	**+**	?	?	?	**+**	**+**	**+**	4
Zhou et al., 2020	**+**	?	?	?	**+**	**+**	**+**	4
Zhu et al., 2017	**+**	?	**?**	**?**	**+**	**+**	**+**	4
Zhu et al., 2016	**+**	?	?	?	**+**	**+**	**+**	4
Zhu Y. et al., 2019	**+**	?	?	?	**+**	**+**	**+**	4

A to G, the 7-item criteria. A, adequate sequence generation; B, concealment of allocation; C, Blinding of participants and personnel; D, Blinding of out-come assessment; E, Incomplete out-come data; F, Selective reporting; G, Other bias; +, low risk of bias; –, high risk of bias; ?, unclear risk of bias.

### Effectiveness

#### Grip strength test

**FIGURE 2:**
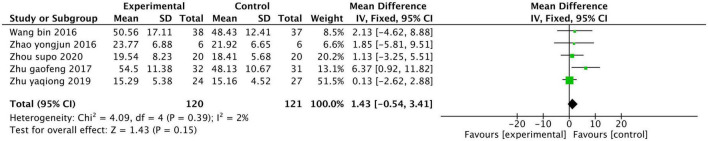
Forest plot of grip strength test.

The grip strength test was conducted in five studies (Wang et al., 2016; Zhao et al., 2016; Zhu et al., 2017; Zhu G. et al., 2019; Zhu Y. et al., 2019; Zhou et al., 2020). Pooled analysis of these five studies indicated that TCE had no significantly greater clinical effects in improving grip strength [MD = 1.43, 95% CI (−0.54, 3.41), *P* = 0.15, I^2^ = 2%; Certainty of evidence: Moderate; Table 3; Figure 2].

**TABLE 3:** Summary of GRADE on evidences of outcomes of traditional Chinese exercise (TCE) for sarcopenia.

Certainty assessment							No of patients		Effect		Certainty	Importance
No. of studies	Study design	Risk of bias	Inconsistency	Indirectness	Imprecision	Other considerations	Trial	Control	Relative (95% CI)	Absolute (95% CI)		
**Grip strength test**												
5	randomized trials	serious^a^	not serious	not serious	not serious	none	120	121	–	MD **1.43 higher** (0.54 lower to 3.41 higher)	⊕⊕⊕○ MODERATE	CRITICAL
**Chair stand test**												
5	randomized trials	serious^a^	not serious	not serious	not serious	none	158	154	–	MD **2.56 higher** (2.09 higher to 3.03 higher)	⊕⊕⊕○ MODERATE	CRITICAL
**Squatting-to-standing test**												
4	randomized trials	serious^a^	not serious	not serious	not serious	none	138	134	–	MD **2.60 higher** (2.25 higher to 2.96 higher)	⊕⊕⊕○ MODERATE	CRITICAL
**6-m gait speed**												
3	randomized trials	serious^a^	not serious	not serious	not serious	none	81	79	–	MD **0.31 higher** (0.30 higher to 0.32 higher)	⊕⊕⊕○ MODERATE	CRITICAL
**Time up and go test**												
3	randomized trials	serious^b^	serious^c^	not serious	not serious	none	68	68	–	MD **1.91 lower** (3.64 lower to 0.19 lower)	⊕⊕○○ LOW	CRITICAL
**Peak torque of the extensors**												
2	randomized trials	serious^a^	not serious	not serious	not serious	none	61	60	–	MD **10.12 higher** (0.90 higher to 19.36 higher)	⊕⊕⊕○ MODERATE	CRITICAL
**Total work of the extensors**												
2	randomized trials	serious^a^	not serious	not serious	not serious	none	61	60	–	MD **113.42 higher** (13.95 higher to 212.89 higher)	⊕⊕⊕○ MODERATE	CRITICAL
**Peak torque of the flexors**												
2	randomized trials	serious^a^	serious^c^	not serious	not serious	none	61	60	–	MD **5.57 higher** (0.60 higher to 10.55 higher)	⊕⊕○○ LOW	CRITICAL
**Total work of the flexors**												
2	randomized trials	serious^a^	serious^c^	not serious	not serious	none	61	60	–	MD **61.79 higher** (10.11 higher to 113.47 higher)	⊕⊕○○ LOW	CRITICAL
**Average power of the flexors**												
2	randomized trials	serious^a^	not serious	not serious	not serious	none	61	60	–	MD **3.25 higher** (0.32 higher to 6.19 higher)	⊕⊕⊕○ MODERATE	CRITICAL
**Berg balance scale**												
4	randomized trials	serious^a^	serious^c^	not serious	not serious	none	107	106	–	MD **1.37 higher** (0.92 higher to 0.83 higher)	⊕⊕○○ LOW	CRITICAL

CI, Confidence interval; MD, Mean difference.

^a^Allocation concealment and blinding were unclear.

^b^Unclear blinding in all studies, allocation concealment in one study.

^c^The statistical test for heterogeneity shows a low *P*-value and the I^2^ is large.

Bold values refers to the MD values.

#### Isokinetic muscle strength test

**FIGURE 3:**
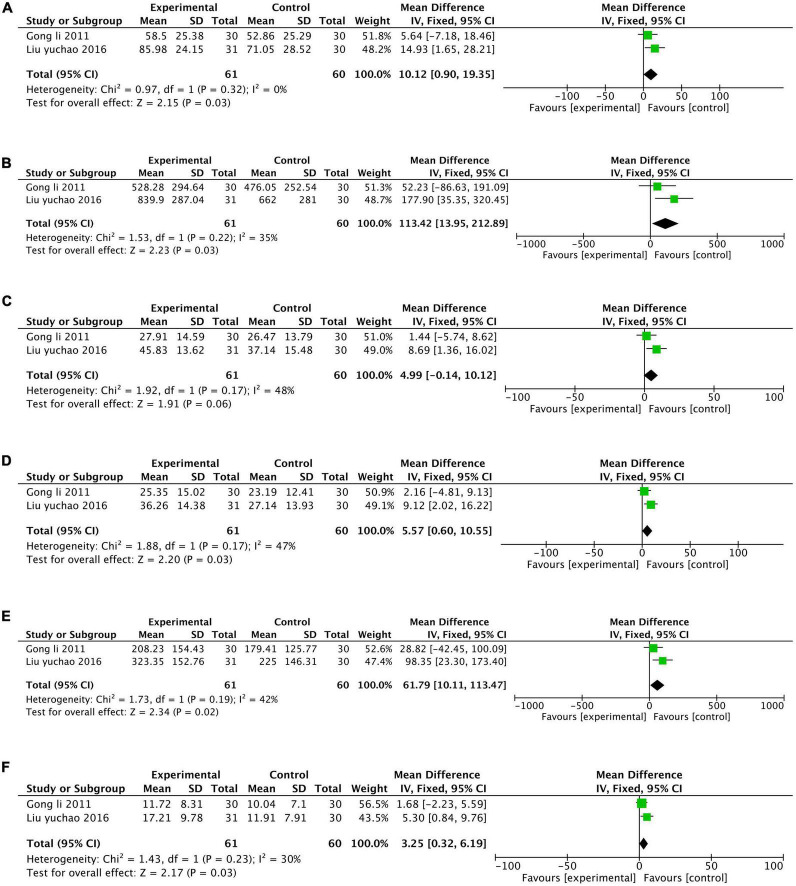
Forest plot of isokinetic muscle strength test: **(A)** the peak torque of the extensors; **(B)** the total work of the extensors; **(C)** the average power of the extensors; **(D)** the peak torque of the flexors; **(E)** the total work of the flexors; **(F)** the average power of the flexors.

Two studies (Gong et al., 2011; Liu et al., 2016) assessed the effect of TCE on the isokinetic muscle strength of participants, and the tests include the Peak torque of the extensors and flexors, the total work of the extensors and flexors, and average power of the extensors and flexors. Meta-analysis indicated that TCE significantly improve participants’ performance in the peak torque of the extensors [MD = 10.12, 95% CI (0.90, 19.35), *P* = 0.03, I^2^ = 0%; Certainty of evidence: Moderate; Figure 3A], the total work of the extensors [MD = 113.42, 95% CI (13.95, 212.89), *P* = 0.03, I^2^ = 35%; Certainty of evidence: Moderate; Figure 3B], the average power of the extensors [MD = 4.99, 95% CI (−0.14, 10.12), *P* = 0.17, I^2^ = 48%; Figure 3C], the peak torque of the flexors [MD = 5.57, 95% CI (0.60, 10.55), *P* = 0.03, I^2^ = 47%; Certainty of evidence: Low; Figure 3D], the total work of the flexors [MD = 61.79, 95% CI (10.11, 113.47), *P* = 0.02, I^2^ = 42%; Certainty of evidence: Low; Figure 3E], and the average power of the flexors [MD = 3.25, 95% CI (0.32, 6.19), *P* = 0.03, I^2^ = 30%; Certainty of evidence: Moderate; Figure 3F].

#### Chair stand test

**FIGURE 4:**
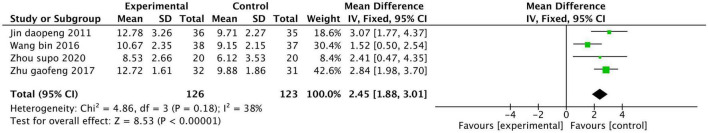
Forest plot of chair stand test.

Meta-analysis of four studies (Jin et al., 2011; Wang et al., 2016; Zhu et al., 2017; Zhu G. et al., 2019; Zhou et al., 2020) showed a significant effect of TCE on chair stand test [MD = 2.45, 95% CI (1.88, 3.01), *P* < 0.00001, I^2^ = 38%; Certainty of evidence: Moderate; Figure 4].

#### Squatting-to-standing test

**FIGURE 5:**
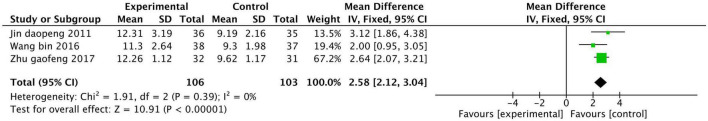
Forest plot of squatting-to-standing test.

Meta-analysis of three studies (Jin et al., 2011; Wang et al., 2016; Zhu et al., 2017; Zhu G. et al., 2019) indicated that TCE could improve participants’ performance in the squatting-to-standing test [MD = 2.58, 95% CI (2.12, 3.04), *P* < 0.00001, I^2^ = 0%; Certainty of evidence: Moderate; Figure 5].

#### 6-m gait speed

**FIGURE 6:**
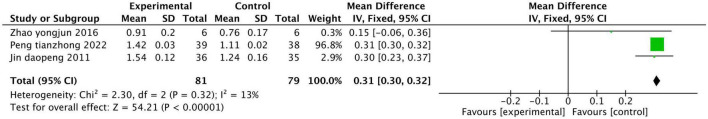
Forest plot of 6-m gait speed.

Meta-analysis of three studies (Jin et al., 2011; Zhao et al., 2016; Peng et al., 2022) showed a significant effect of TCE in improving the 6-m gait speed of participants [MD = 0.31, 95% CI (0.30, 0.32), *P* < 0.00001, I^2^ = 13%; Certainty of evidence: Moderate; Figure 6].

#### Time up and go test

**FIGURE 7:**
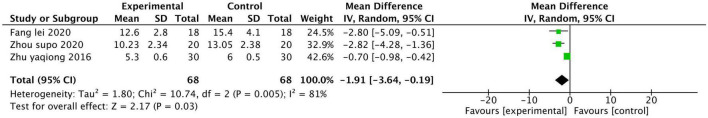
Forest plot of time up and go test.

Pooled analysis of three studies (Zhu et al., 2016; Fang et al., 2020; Zhou et al., 2020) showed that TCE significantly improved the preference of participants in the Time Up and Go Test [MD = −1.91, 95% CI (−3.64, −0.19), *P* = 0.03, I^2^ = 81%; Certainty of evidence: Low; Figure 7].

#### Balance function

**FIGURE 8:**
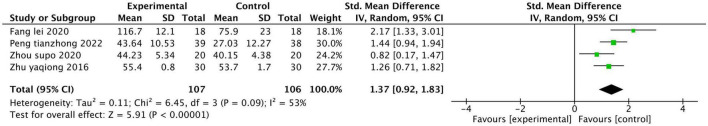
Forest plot of Berg Balance Scale.

Meta-analysis of four studies (Zhu et al., 2016; Fang et al., 2020; Zhou et al., 2020; Peng et al., 2022) showed a significant effect of TCE in improving balance function according to Berg balance scale [SMD = 1.37, 95% CI (0.92, 1.83), *P* < 0.00001, I^2^ = 53%; Certainty of evidence: Low; Figure 8]. The Biodex system was used to assess the balance function in 1 study (Liu et al., 2014), and the results indicated that Yijinjing had significantly greater clinical effects in improving balance function with open eyes (*P* < 0.05).

#### Adverse events

Side effects of TCE were evaluated in two studies (Liu et al., 2012; Liu et al., 2014), but adverse events were not observed in these two studies.

## Discussion

This study is the first meta-analysis assessing the efficacy of TCE for sarcopenia. Thirteen studies with 718 subjects were identified. The methodological quality of included RCTs was moderate totally. The quality of the evidence of primary outcomes was low to moderate according to the GRADE profiler. The main findings of the present study were that the TCE had a greater clinical effect in improving the severity of sarcopenia compared with no training or health education.

In this study, the primary outcomes of TCE for sarcopenia were muscle strength and physical function, since the decrease in muscle strength and physical function were the primary problem caused by sarcopenia (Hanach et al., 2019). The results of the pooled analysis indicated that TCE had no significantly greater clinical effects in improving grip strength, but had significantly greater clinical effects in physical function according to various outcomes including chair stand test, squatting-to-standing test, 6-m gait speed, Time Up and Go Test, peak torque of the extensors, total work of the extensors, peak torque of the flexors, total work of the flexors, the average power of the flexors, and balance function. In Western society, as many as 42% of individuals under 60 years of age have difficulties performing the activities of daily life, 15–30% report being unable to lift or carry 10 pounds or more, and more than 30% are confronted with physical disabilities (Zhou et al., 2019). Therefore, the positive results of TCE in improving physical function have great clinical significance.

Traditional Chinese exercise were formed by the concept of viewing the situation as a whole, the Five-Zang manifestation theory and meridian doctrine as theoretical guidance, and body movement as presentation. They are aimed to enhance fitness and prevent and treat diseases (Yang et al., 2021). TCE may be used to delay sarcopenia by regulating the synthesis and degradation of muscle-related proteins, replenishing, nutrients, promoting blood circulation, and eliminating inflammation (Colleluori and Villareal, 2021). The potential mechanism of TCE for enhancing muscle strength and physical function is related to the activation of key signaling pathways (Liu et al., 2021). After high-intensity interval static exercise, the PGC-1α/FNDC5/UCP1 signaling pathway was activated, PGC-1α was up-regulated, mitochondria increased, muscle fiber thickening was observed, and the skeletal muscle atrophy state was improved in aging rats (Liu et al., 2021). Compared with general exercise, TCE are more like gymnastic exercise consisting of various components such as endurance, resistance, balance, flexibility, breathing, and meditation, which emphasize the appropriate form and intensity of exercise, resulting in a better response (Villareal et al., 2017; Colleluori and Villareal, 2021).

The major strength of the current systematic review is that it has adhered to appropriate systematic review guidelines. However, there are also some limitations. First, some methodological limitations exist in the primary studies. Only one study (Fang et al., 2020) reported the concealment of allocation. Trials with adequate concealment had an average of 18% less “beneficial” effect than trials with inadequate or unclear concealment of allocation (Cumpston et al., 2019). Performance bias and detection bias can be effectively avoided by the use of blinding. However, some studies were unable to be blinded because participants have a high degree of understanding of TCE moves. Only two studies reported the blinding of outcome assessment. Second, formal pretrial sample size calculation was not conducted in most clinical trials and the majority of the included trials had relatively small sample sizes. Trials with insufficient statistical power may induce the high a risk of overestimating therapeutic efficacy (Kjaergard et al., 2001). Third, no study describes the duration of follow-up, making it difficult to assess the long-term efficacy of TCE treatment for sarcopenia. Fourth, we only searched for papers published in Chinese or English databases, thus the eligible studies published in other languages may be left out, which may limit the generalizability of the findings. Fifth, a statement published in September 2004 requiring that all clinical trials must be registered to be considered for publication (De Angelis et al., 2004). The transparency of clinical trials would be improved with registration, which would ultimately strengthen the validity and value of the scientific evidence base (Wang et al., 2019a). However, none of the included studies had been registered formally.

The finding from the present systematic review revealed that TCE may be beneficial for sarcopenia patients. However, as the low-quality studies included cannot be reproduced, there is a need for conducting further rigorous RCTs on TCE for sarcopenia. Recommendations for further research are as follows: (1) the protocol of further clinical trials should be registered in the international clinical trials registry platform prospectively, and should follow the requirement of the Clinical Trial Data Sharing Statement (Taichman et al., 2016) by the International Committee of Medical Journal Editors; (2) the quality of study designs including randomization, allocation concealment, and blinding should be improved. CONSORT statement (Moher et al., 2009) should be applied throughout the whole process of the study including trial design, reporting, and publication; (3) international cooperation should be conducted in further studies to complete more qualified studies and ensure generalizability of research findings; (4) greater consistency in outcome measures should be warranted; (5) adequate sample size plays an important positive role in improving the methodologic quality, intervention effects, and publication bias (Kjaergard et al., 2001; Moher et al., 2009). Thus, it is necessary to conduct formal pretrial sample size calculations in further studies.

The significance of the present systematic review possibly lies in the following aspects: (1) to reveal current problems in the treatment of sarcopenia and identify areas worthy of improvement and development in the future (Chan et al., 2012). Several studies have reported the effectiveness of TCE in the treatment of sarcopenia, however, no previous study has evaluated the quality of this evidence. (3) to report a specific area of Traditional Chinese Medicine in the English language as these experiences are not readily accessible to western clinicians because of language barriers (Wang et al., 2019b).

## Conclusion

The present finding indicated that TCE provided statistically significant benefits for sarcopenia. Therefore, the findings of the present systematic review, at least to a certain extent, provided supporting evidence for the routine use of TCE for sarcopenia.

## Data availability statement

The original contributions presented in this study are included in the article/supplementary material, further inquiries can be directed to the corresponding authors.

## Author contributions

KN, Y-LL, X-ZZ, and QQ performed conceptualization. KN and Y-LL contributed to the formal analysis, visualization, methodology, and writing the original draft. KN, Y-LL, FY, and YW performed data curation. KN, FY, YW, X-ZZ, and QQ performed writing—review and editing. All authors contributed to the article and approved the submitted version.
